# Comparison of asthma phenotypes in OVA-induced mice challenged via inhaled and intranasal routes

**DOI:** 10.1186/s12890-019-1001-9

**Published:** 2019-12-10

**Authors:** Dong Im Kim, Mi-Kyung Song, Kyuhong Lee

**Affiliations:** 1 National Center for Efficacy evaluation for Respiratory disease product, Jeonbuk Department of Inhalation Research, Korea Institute of Toxicology, 30 Baehak1-gil, Jeongeup, Jeollabuk-do 56212 Republic of Korea; 2 Department of human and environmental toxicology, University of Science & Technology, Daejeon, 34113 Republic of Korea

**Keywords:** Allergic asthma, Animal model, Asthma phenotypes, Challenge routes, Ovalbumin sensitization

## Abstract

**Background:**

The respiratory system is exposed to various allergens via inhaled and intranasal routes. Murine models of allergic lung disease have been developed to clarify the mechanisms underlying inflammatory responses and evaluate the efficacy of novel therapeutics. However, there have been no comparative studies on differences in allergic phenotypes following inhaled vs. intranasal allergen challenge. In this study, we compared the asthmatic features of mice challenged via different routes following allergen sensitization and investigated the underlying mechanisms.

**Methods:**

To establish ovalbumin (OVA)-induced allergic asthma models, BALB/c mice were sensitized to 20 μg OVA with 1 mg aluminum hydroxide by the intraperitoneal route and then challenged by inhalation or intranasal administration with 5% OVA for 3 consecutive days. Cellular changes and immunoglobulin (Ig) E levels in bronchoalveolar lavage fluid (BALF) and serum, respectively, were assessed. Histological changes in the lungs were examined by hematoxylin and eosin (H&E) and periodic acid Schiff (PAS) staining. Levels of T helper (Th)2 cytokines including interleukin (IL)-4, -5, and -13 in BALF and epithelial cytokines including IL-25 and -33 in BALF and lung tissues were measured by enzyme-linked immunosorbent assay and western blotting. Airway hyperresponsiveness (AHR) was evaluated by assessing airway resistance (Rrs) and elastance (E) via an invasive method.

**Results:**

OVA-sensitized and challenged mice showed typical asthma features such as airway inflammation, elevated IgE level, and AHR regardless of the challenge route. However, H&E staining showed that inflammation of pulmonary vessels, alveolar ducts, and alveoli were enhanced by inhaled as compared to intranasal OVA challenge. PAS staining showed that intranasal OVA challenge induced severe mucus production accompanied by inflammation in bronchial regions. In addition, Th2 cytokine levels in BALF and AHR in lung were increased to a greater extent by inhalation than by intranasal administration of OVA. Epithelial cytokine expression, especially IL-25, was increased in the lungs of mice in the inhaled OVA challenge group.

**Conclusion:**

OVA-sensitized mice exhibit different pathophysiological patterns of asthma including expression of epithelial cell-derived cytokines depending on the OVA challenge route. Thus, some heterogeneous phenotypes of human asthma can be replicated by varying the mode of delivery after OVA sensitization.

## Background

Asthma is a chronic airway disease induced by exposure to environmental triggers and is characterized by airway inflammation, airway hyperresponsiveness (AHR), elevated immunoglobulin (Ig) E level, and airway remodeling (e.g., subepithelial and airway wall fibrosis, goblet cell hyperplasia/metaplasia, increased smooth muscle mass, and increased vascularity) accompanied by clinical symptoms such as wheezing, shortness of breath, chest tightness, cough, and restricted airflow [1–3]. Asthma affects people of all ages, with a higher prevalence in male children and female adults. Elderly patients with asthma are at a higher risk for morbidity and mortality [4, 5]. The asthma phenotype is characterized according to clinical parameters, physiological criteria, and environmental triggers [6]. However, the phenotype varies between individuals and over time in a single individual [7]. At present, there is no standard method for distinguishing between different asthma phenotypes [6].

Although clinical studies are the best approach for investigating human asthma, these can be limited by ethical considerations. Animal models have therefore been used as an alternative tool for studying human asthma development and progression [3]. Mice have many advantages including a well-characterized immune system, the availability of transgenic animals, and a large array of reagents for analyzing cellular and molecular responses [8, 9]. Various approaches have been used to induce asthma in mice, including different mouse strains, allergens, and administration routes [10, 11]. Ovalbumin (OVA)-sensitized and challenged BALB/c mice are widely used as an asthma model and are characterized by high levels of serum IgE, airway inflammation, epithelial hypertrophy, goblet cell hyperplasia, and AHR, which are similar to the features observed in human asthma. However, the pattern of lung inflammation and its distribution in the lower airway of mice differs from those in humans due to species differences in lung branching [12]. To establish an animal model of asthma that more closely reflects the human disease, asthma phenotypes induced by allergen administration routes other than intraperitoneal or subcutaneous injection have been investigated [13–15], such as inhaled or intranasal delivery, which occurs in human asthma [16, 17]. However, there have been no studies analyzing the pathology of allergic asthma by these various challenge routes in animal model of asthma. We hypothesized that allergen challenge routes can affect different pathophysiological asthma pattern and heterogeneous phenotypes of human asthma can be replicated by changing the mode of delivery after allergen sensitization.

We addressed this in the present study by comparing the pathophysiology of asthma induced in OVA-induced BALB/c mice by inhalation or intranasal administration. We evaluated changes in asthmatic phenotype including airway inflammation, AHR, histological features, and levels of IgE and inflammatory and epithelial cytokines.

## Methods

### Animals

Female Balb/c mice (Orient Bio Ltd., Seongnam, Korea), weighting 14.9~17.3 g, were housed in light (12 h light: 12 h dark cycle) and temperature-controlled (22 ± 3 °C) rooms. Throughout the experiments, they had free access to standard laboratory chow and were provided with tap water ad libitum. They were used for the murine models after 8 days of acclimation with no adverse clinical signs and normal weight gain. The experiments were performed in accordance with protocols approved by the Institutional Animal Care and Use Committee of the Korea Institute of Toxicology (no. 1907-0244). The study consisted of 5 groups (each group, *n* = 5): naive control group, inhaled saline group, intranasal saline group, inhaled OVA group, and intranasal OVA group. The mice were randomly divided into 5 weight-matched experimental groups using the Pristima System (Version 7.3; Xybion Medical Systems Corporation, USA).

### Sensitization and challenge protocol for allergic asthma

Mice in the naïve control group received no treatment for the duration of the experiment. Mice in the inhaled saline group were sensitized intraperitoneally with 200 μl saline on days 1 and 8. On days 15, 16, and 17 after initial sensitization with saline, mice were nebulized with saline for 30 min in a whole-body exposure chamber. Mice in the intranasal saline group were intraperitoneally sensitized as inhaled saline group and then intranasally instilled with 40 μl saline on days 15, 16, and 17. Mice in the inhaled OVA group were intraperitoneally sensitized on days 1 and 8 with 20 μg OVA (Sigma-Aldrich, St. Louis, MO) emulsified in 1 mg aluminum hydroxide (Thermo Fisher Scientific, Waltham, MA) in a total volume of 200 μl. On days 15, 16, and 17 after initial sensitization with OVA, mice were administered aerosolized 5% (v/v) OVA by inhalation for 30 min in a whole-body exposure chamber. Mice in the intranasal OVA group were intraperitoneally sensitized as the inhaled OVA group and then intranasally instilled with 20 μg OVA on days 15, 16, and 17. For intranasal instillation, mice were anesthetized using inhaled anesthesia. Prior to instillation, isoflurane was delivered into induction chamber using small animal portable anesthesia systems (L-PAS-02, LMSKOREA, Inc., Seongnam, Korea) equipped with isoflurane vaporizer. And then, mice were exposed to 2.5% isoflurane delivered in O_2_ (2 L/min) within induction chamber until a sleep-like state was reached. Mice receiving isoflurane anesthesia were removed from the induction chamber and instillation was performed immediately on board. Intranasal administration of challenge dose (saline or 20 μg OVA diluted in 40 μl saline) was performed by pipetting onto the outer edge of nose of the mice. After instillation, mice moved, fully recovered and transferred to their cage.

Twenty-four hours after the last challenge with OVA, AHR were assessed. And 48 h after the last challenge with OVA, mice were euthanized with an overdose of isoflurane and continuously exposed until 1 min after breathing stops. The sample collections for analysis were performed in sacrificed animals. Lung inflammation, serum IgE production, and protein levels of inflammatory and epithelial cell-derived cytokines were assessed.

### Measurement of body and organ weights

The body weight of mice was measured on days 1, 3, 8, 10, 15, 17, and 19 after initial sensitization with OVA. On day 19, mice were sacrificed and left lung and spleen weights were recorded.

### Bronchoalveolar lavage fluid (BALF) preparation

At 48 h after the final OVA challenge, mice were anesthetized, the left lung was ligated and the right lungs were gently lavaged three times via the tracheal tube with a total volume of 0.7 ml phosphate buffered saline (PBS). The total number of cells in the collected BALF was counted with a NucleoCounter (NC-250; ChemoMetec, Gydevang, Denmark). For differential cell counts, BALF cells smears were prepared using Cytospin (Thermo Fisher Scientific) and were stained with Diff-Quik solution (Dade Diagnostics, Aguada, Puerto Rico). The different cell types were counted (*n* = 200/slide). BALF was immediately centrifuged at 2000 rpm for 5 min, and the collected supernatant was stored at − 70 °C until measurement of cytokine levels by enzyme-linked immunosorbent assay (ELISA).

### Measurement of cytokine levels

Interleukin (IL)-4, -5, -13, -25, and -33 levels in BALF were quantified by ELISA using commercial kits (Thermo Fisher Scientific) according to the manufacturer’s protocol. The sensitivity for IL-4, -5, -13, -25, and -33 assays were 0.32, 3.3, 2.8, 0.9 and 11.2 pg/ml, respectively. The intra and inter assay coefficients of variation for IL-4, -5, -13, -25, and -33 were 7.1 7.9, 5, 6.5, and 6.7%, and 10, 4.6, 5, 8.8, and 3.6%, respectively.

### Measurement of total serum IgE level

At 48 h after the last OVA challenge, mice were anesthetized with isoflurane, and blood samples were obtained from the abdominal aorta and centrifuged at 3000 rpm for 10 min. Total IgE level in serum was determined by ELISA using purified rat anti-mouse IgE and biotinylated rat anti-mouse IgE as capture and detection antibodies, respectively (both from BD Pharmingen, San Diego, CA, USA). Standards were prepared using purified mouse IgE κ isotype control (BD Pharmingen). Optical density was determined by measuring the absorbance at 450 nm.

### Histological analysis

At 48 h after the last OVA challenge, mice were sacrificed for histological examination. Lung tissue was removed and fixed in 10% (v/v) neutral-buffered formalin then dehydrated, embedded in paraffin, and cut into 4-μm sections that were deparaffinized with xylene and then stained with hematoxylin and eosin (H&E) and periodic acid Schiff (PAS) (both from Sigma-Aldrich). Stained sections were analyzed under a light microscope (Axio Imager M1; Carl Zeiss, Oberkochen, Germany). The degree of lung inflammation and goblet cell hyperplasia was scored on a subjective scale of 0 to 4 as previously described [18]. Briefly, to score the inflammatory cell infiltration in the intraluminal, alveolar, peribronchial, and perivascular regions, cell counts were performed blind based on five point grading system for the following features: 0: normal, 1: few cells, 2: a ring of inflammatory cells 1 cell layer deep; 3: a ring of inflammatory cells 2-4 cells deep, 4: a ring of inflammatory cells of > 4 cells deep. For the quantification of goblet cells in the bronchi and bronchioles, five point grading system was used, 0: < 0.5% PAS positive cells, 1: < 25%, 2: 25-50%, 3: 50-75% and 4: > 75%. Five fields were counted for each slide and mean score was calculated from five animals. Quantification of the PAS-positive goblet cells was expressed as the number of PAS-positive cells per mm of basement membrane to correct for airway size [19].

### Measurement of AHR

To measure lung function, mice were anesthetized 24 h after the last OVA challenge with 80 mg/kg alfaxalone and then tracheotomized using a computer-controlled animal ventilator (eSpira; EMMS, Edinburgh, UK). Lungs were inflated one successive time to total lung capacity (TLC) at 20 cm of H_2_O. Airway resistance (Rrs) and elastance (E) of the whole respiratory system were measured using a 1.2-s single frequency sinusoidal oscillation at a frequency of 150 breaths/min, termed a ‘snapshot150’ maneuver. Rrs and E values were recorded at baseline and following a 10 s exposure to an increasing concentration of methacholine (0, 6.125, 12.5, 25, and 50 mg/ml). The absolute values of 13 snapshot150 maneuvers were calculated.

### Western blot analysis

Lung tissues were homogenized and lysed in RIPA buffer (Thermo Fisher Scientific) with a protease inhibitor cocktail. Protein concentrations were determined using Bradford reagent (Bio-Rad Laboratories, Hercules, CA). Samples were loaded onto a SDS-PAGE gel. After electrophoresis at 120 V for 90 min, proteins were transferred to polyvinylidene difluoride membranes (Bio-Rad Laboratories) at 250 mA for 90 min by a wet transfer method. Nonspecific sites were blocked with 5% non-fat dry milk in Tris-buffered saline Tween 20 (25 mmol/l Tris pH 7.5, 150 mmol/l NaCl, 0.1% Tween 20) for 1 h, and the blots were then incubated overnight at 4 °C with an anti-IL-25 (Biolegend Inc., Pacific Heights Blvd, San Diego, CA) antibody, anti-IL-33 (Thermo Fisher Scientific) antibody, and anti-actin (Santa Cruz Biotechnology, Santa Cruz, CA) antibody as a reference protein. Anti-rat (Santa Cruz Biotechnology), anti-rabbit, or anti-mouse horseradish peroxidase-conjugated-IgG (Cell Signaling Technology, Beverly, MA) was used to detect binding of antibodies. The binding of the specific antibody was visualized using iBright CL1000 imaging system (Thermo Fisher Scientific) after treating with enhanced chemiluminescence system reagents (Thermo Fisher Scientific). Densitometric analysis was performed on the relative intensity of each band using the iBright CL1000 image software. For the quantification of specific bands, the square with the same size was drawn around each band to measure the density and then the value was adjusted by the density of the background near that band. The results of densitometric analysis were expressed as a relative ratio of the target protein to reference protein. The relative ratio of the target protein of control group is arbitrarily presented as 1.

### Statistical analysis

Except Rrs and E values data, all statistical analyses were performed using SigmaPlot v.12 software (Systat Software, San Jose, CA, USA). Data are expressed as mean ± SD. Test for normality was performed by Shapiro-Wilk test. Statistical multiple comparisons were performed by one-way analysis of variance followed by Dunnett test. Data of both Rrs and E values were expressed as mean ± SEM and were analyzed using a two-way ANOVA with Bonferroni’s Multiple Comparison Test for repeated measurements. Comparisons between two groups were carried out with the t-test for paired variables or Mann-Whitney U test for unpaired variables. *p* < 0.05 was considered statistically significant.

## Results

### Changes in body and organ weights

**Fig. 1:**
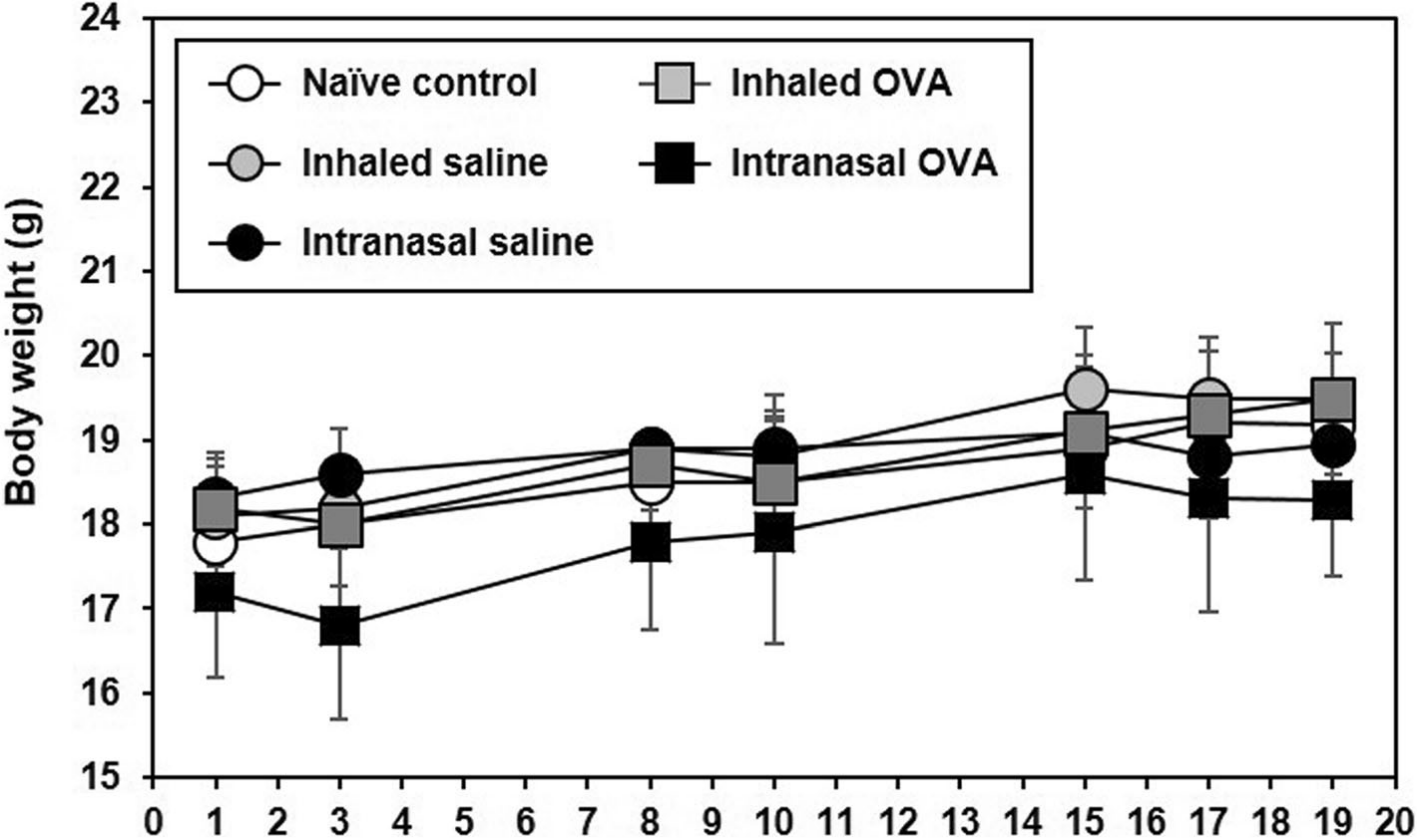
Changes in body weight of control and OVA-sensitized and challenged mice

**Fig. 2:**
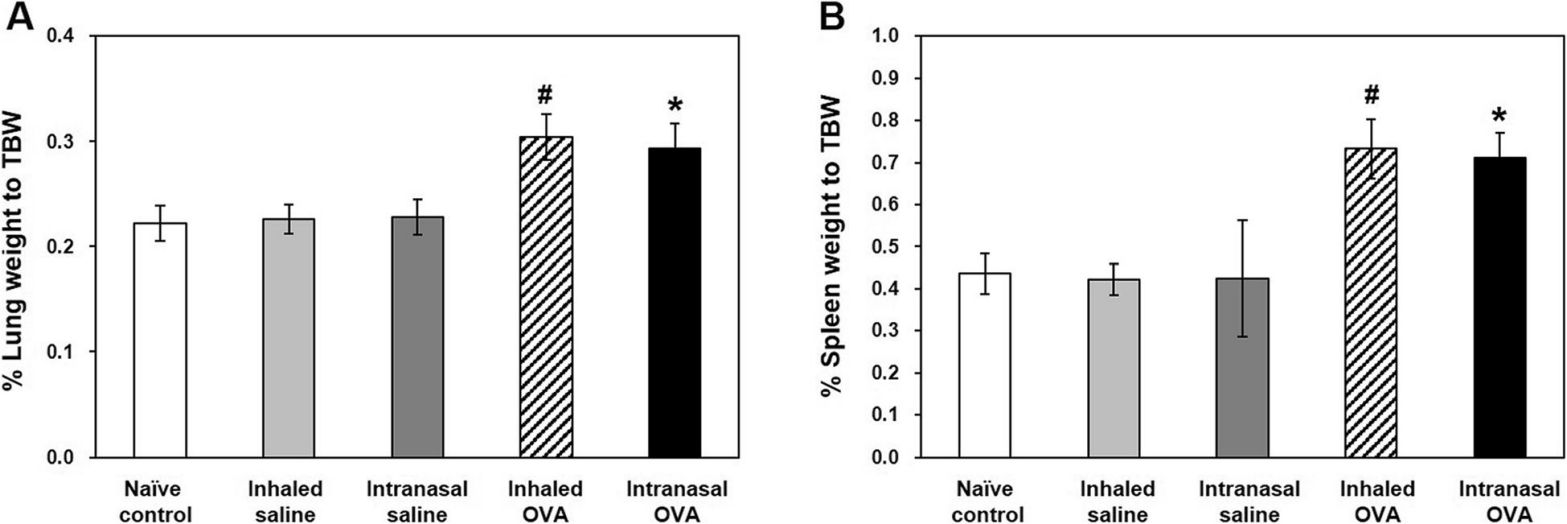
Changes in relative lung and spleen weights of control and OVA-sensitized and challenged mice. **a**, **b** Relative lung (**a**) and spleen (**b**) weights were calculated using the following formula: relative organ weight = organ weight (g)/terminal body weight (g) × 100%. Bars represent the mean ± SD from five mice per group. ^#^*p* < 0.05 vs. inhaled saline, ^*^p < 0.05 vs. intranasal saline

The body weight of mice remained at a constant level during the experimental period, and no significant differences were observed between groups (Fig. 1). Relative lung (*p* < 0.05; Fig. 2a) and spleen (*p* < 0.05; Fig. 2b) weights were increased in inhaled OVA group and intranasal OVA group as compared with inhaled saline group and intranasal saline group, respectively, but there was no significant difference between both OVA groups.

### Cellular changes in BALF

**Fig. 3:**
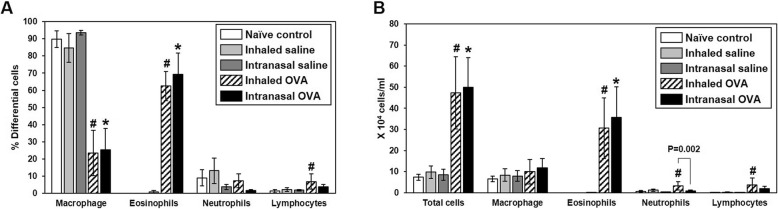
Cellular changes in BALF. BALF cells were collected and analyzed 48 h after the last OVA challenge. Percentages of differential cells in BALF from control and OVA-sensitized and challenged mice (**a**). Total and differential cells in BALF (**b**). Bars represent the mean ± SD from five mice per group. ^#^*p* < 0.05 vs. inhaled saline, ^*^p < 0.05 vs. intranasal saline

To determine whether the lung inflammatory response varies according to the OVA challenge route, we analyzed inflammatory cells in BALF of OVA-induced mice. Following OVA sensitization, inhaled and intranasal OVA challenge resulted in significant increases in eosinophil population in BALF as compared to mice in the each control group (*p* < 0.05; Fig. 3a), although there was no significant difference between them. The number of total cells and eosinophils in BALF was significantly elevated as compared to mice in the each control group (*p* < 0.05; Fig. 3b). Especially, inhaled OVA challenge statistically induced the number of neutrophils and lymphocytes as compared to mice in the inhaled saline control group. Also, the number of neutrophils was higher in the inhaled as compared to the intranasal OVA group (*p* = 0.002; Fig. 3a). These results indicate that OVA sensitization and challenge induces eosinophil-dominant allergic lung inflammation in mice and allergen challenge routes can affect different asthmatic pattern.

### Total serum IgE level

**Fig. 4:**
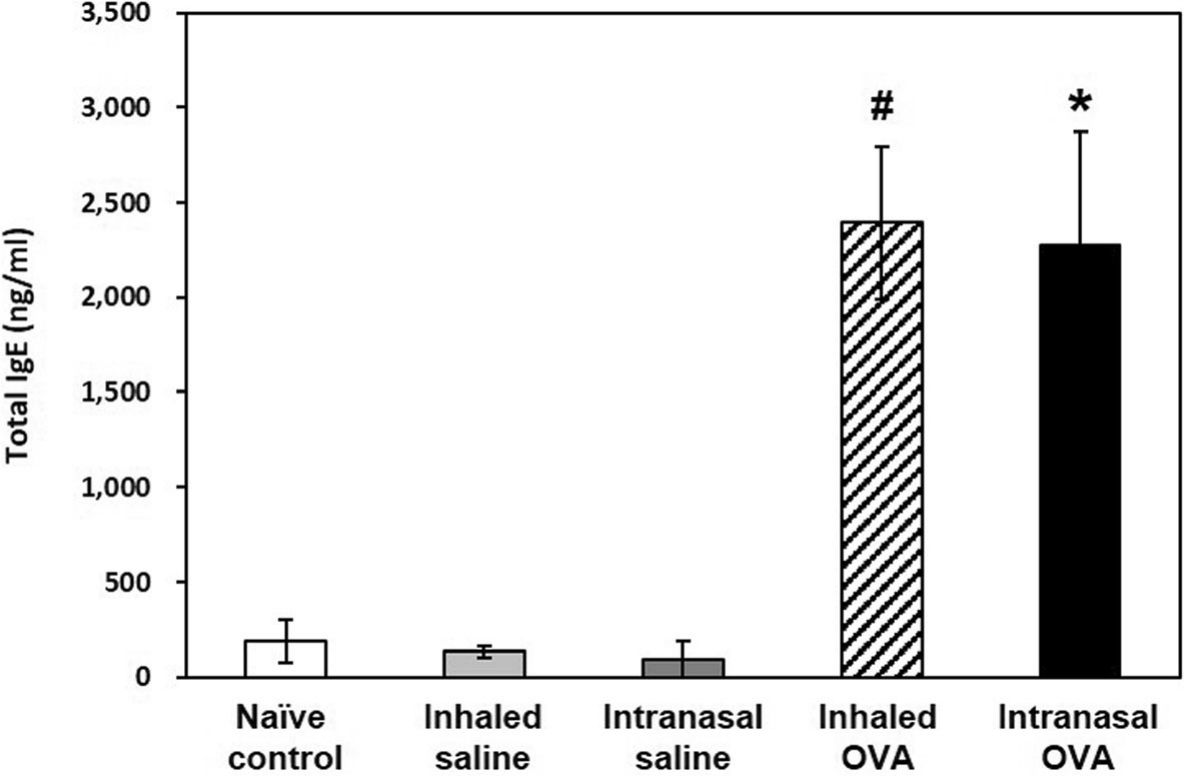
Serum IgE levels in control and OVA-sensitized and challenged mice. Serum samples from all groups were collected 48 h after the last OVA challenge. Bars represent the mean ± SD from five mice per group. ^#^*p* < 0.05 vs. inhaled saline, ^*^p < 0.05 vs. intranasal saline

We next investigated whether OVA sensitization and challenge induces IgE-mediated allergic asthma and whether serum IgE is influenced by the OVA challenge route. Total IgE in serum was elevated in both OVA-induced groups relative to the each control group (*p* < 0.05; Fig. 4), although there was no difference according to the OVA challenge route.

### Histological changes in lung

**Fig. 5:**
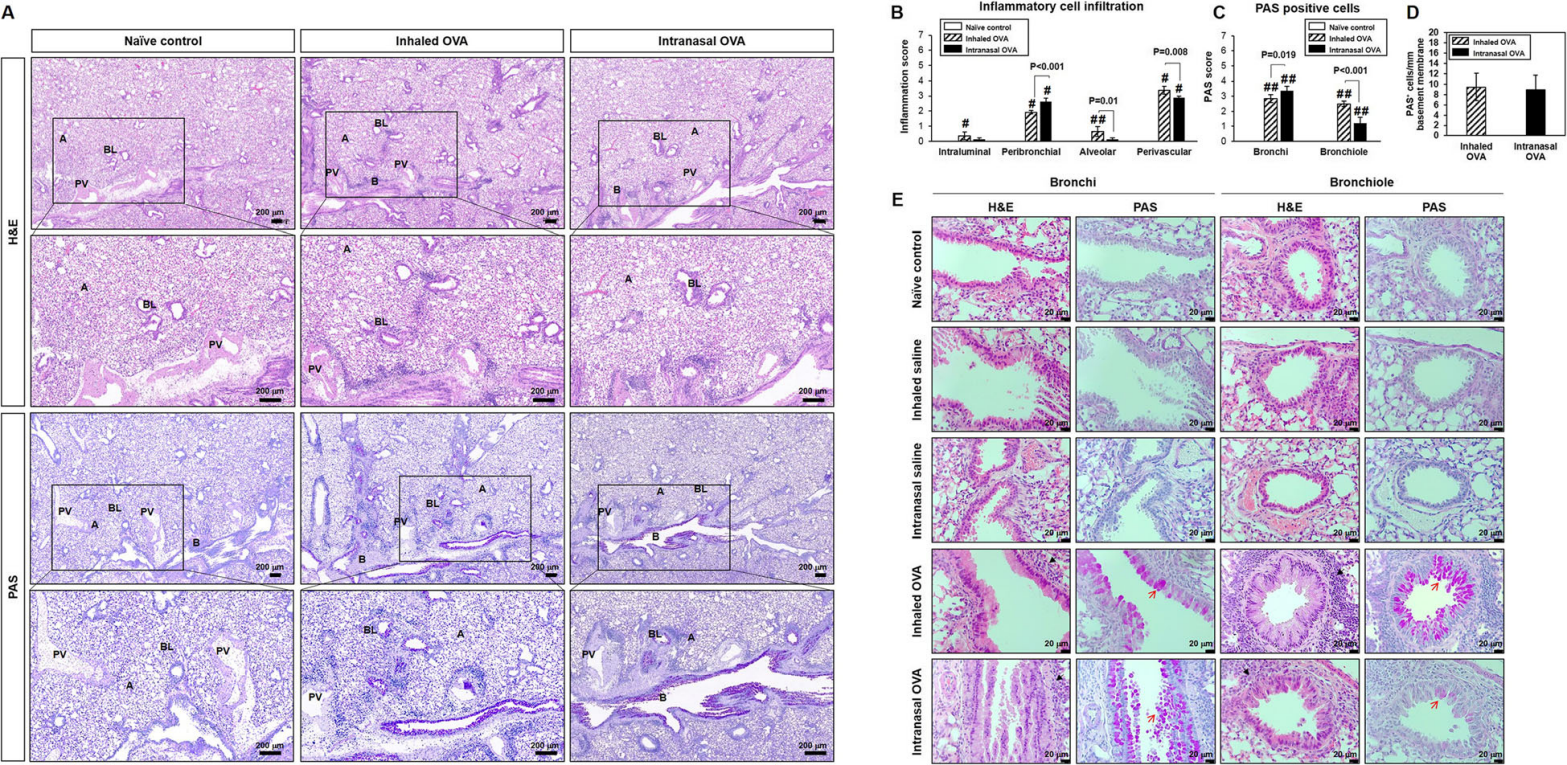
Infiltration of inflammatory cells and mucus secretion in lung tissue from control and OVA-sensitized and challenged mice. Representative H&E- and PAS-stained sections of lung and higher magnifications (**a**). Histological scoring of inflammatory cell infiltration (**b**) and goblet cells (**c**). Number of PAS^+^ cells were counted and normalized by area of basement membrane (**d**). Representative H&E- and PAS-stained sections in bronchi and bronchiole (**e**). Black and red arrows indicate inflammatory cells and goblet cells. B; bronchi, BL; bronchiole, A; alveolar, PV; perivascular. Bars represent the mean ± SD from five mice per group. ^#^*p* < 0.05 vs. naïve control

We carried out a histological analysis to identify the pathological features of OVA-induced allergic lung inflammation and mucus production (Fig. 5, Table 1). The typical pathological features of allergic asthma were observed in both OVA-induced groups as compared to control groups by H&E staining. The inhaled OVA group showed eosinophilic infiltration in pulmonary vessels, alveolar ducts, and whole lung alveoli (Fig. 5a). In contrast, the intranasal OVA group mainly showed inflammation in peribronchial regions (Fig. 5a). The observations were further confirmed by histological scoring of the inflammatory cells infiltration on the route of OVA challenge (Fig. 5b). PAS staining and the number of PAS-positive cells per mm basement membrane was markedly increased in both OVA-induced mice as compared to control group (Fig. 5c, Fig. 5d). The total amount of mucus production was similar between the two OVA-induced mice groups (Fig. 5d). Interestingly, mucus production in intranasal OVA group was mainly observed in bronchi epithelium as compared to the inhaled route (Fig. 5e). These results indicate that asthmatic histopathology in the lungs is differentially induced depending on the mode of OVA challenge.

**Table 1:** Histologic scores in lungs of OVA-induced allergic asthma mice

Group	Naïve control	Inhaled saline	Intranasal saline	Inhaled OVA	Intranasal OVA
Number examined	5	5	5	5	5
Infiltrate, mainly eosinophilic cells, pulmonary vessel/alveolar duct/alveoli					
Moderate	0	0	0	4	5
Severe	0	0	0	1	0
Total number affected	0	0	0	5	5
Inflammation, granulomatous, peribronchial					
Minimal	0	0	0	0	1
Slight	0	0	0	0	1
Total number affected	0	0	0	0	2
Metaplasia, mucous cell confirmed by PAS stain					
Slight	0	0	0	3	1
Moderate	0	0	0	2	4
Total number affected	0	0	0	5	5

### T helper (Th)2 cytokine levels in BALF and AHR in lung

**Fig. 6:**
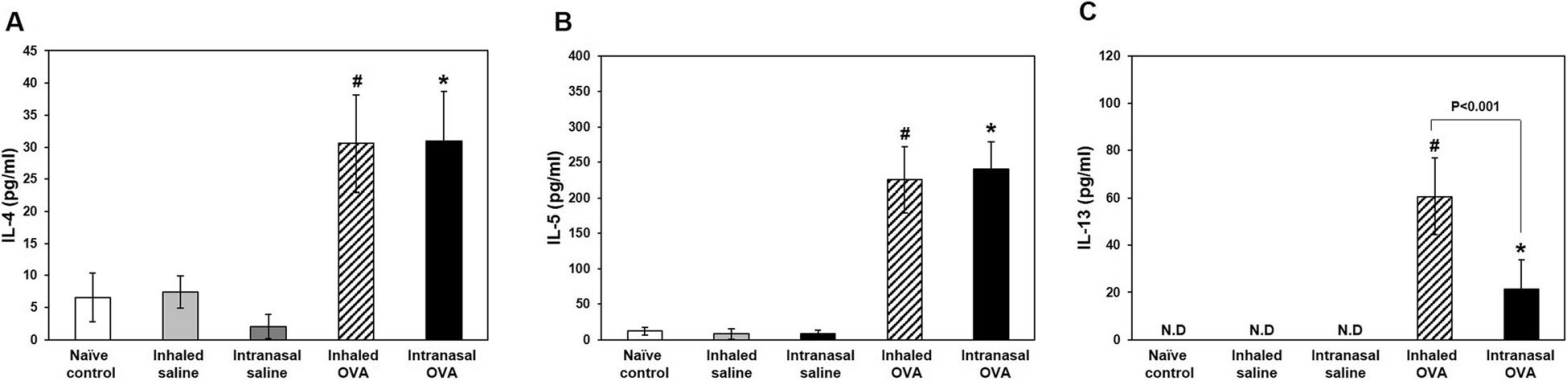
Th2 cytokine levels in BALF. Enzyme immunoassay of IL-4 (**a**), IL-5 (**b**), and IL-13 (**c**) levels. Bars represent mean ± SD (*n* = 5/group). ^#^*p* < 0.05 vs. inhaled saline, ^*^p < 0.05 vs. intranasal saline

**Fig. 7:**
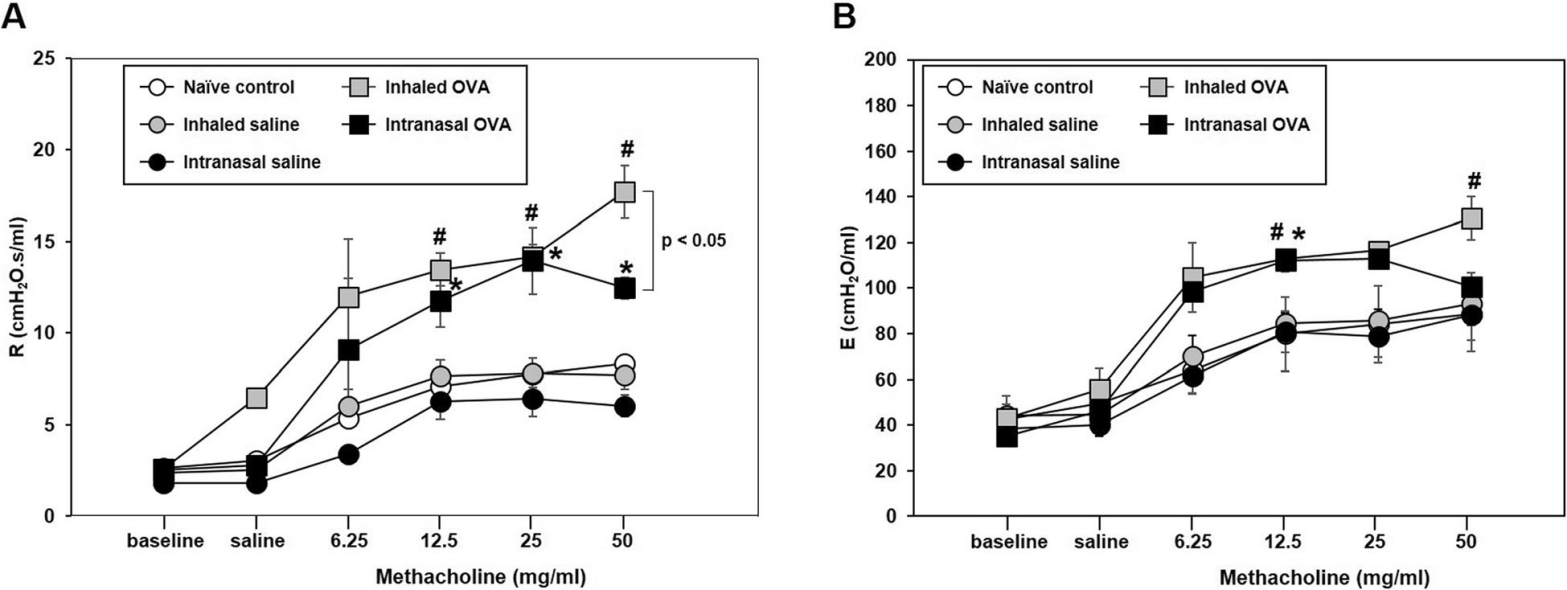
Changes in airway responsiveness according to methacholine concentration in control and OVA-sensitized and challenged mice. **a** Respiratory system resistance (Rrs) and (**b**) elastance (E) was measured 24 h after the last OVA challenge in mice. Bars represent the mean ± SEM (*n* = 5/group). ^#^*p* < 0.05 vs. inhaled saline, ^*^p < 0.05 vs. intranasal saline

Th2 cytokines play important roles in allergic asthma during airway remodeling and the development of airway resistance [20, 21]. We assessed Th2 cytokine levels in BALF and AHR including airway resistance (Rrs) and elastance (E) in the lungs following OVA challenge and found that Th2 cytokine IL-4, -5, and -13 levels in BALF were higher in both OVA-induced groups than in each control mice (*p* < 0.05; Fig. 6); moreover, the levels of Th2 cytokines especially IL-13 were higher in the inhaled as compared to the intranasal OVA group (*p < 0*.001; Fig. 6c). Consistent with these observations, Rrs value were significantly increased by 12.5, 25, and 50 mg/ml methacholine treatment in both OVA-induced groups as compared to the each control group. The Rrs of 50 mg/ml methacholine treatment were statistically higher in the inhaled OVA group than those in the intranasal OVA group (*p* < 0.05; Fig. 7a). In addition, E value in inhaled OVA groups were significantly increased by 12.5 and 50 mg/ml methacholine treatment as compared to the inhaled saline control group (*p* < 0.05; Fig. 7b). These results suggest that OVA-induced asthmatic responses are more potently induced by inhaled rather than intranasal OVA challenge.

### Epithelial cytokine levels in BALF and lung tissues

**Fig. 8:**
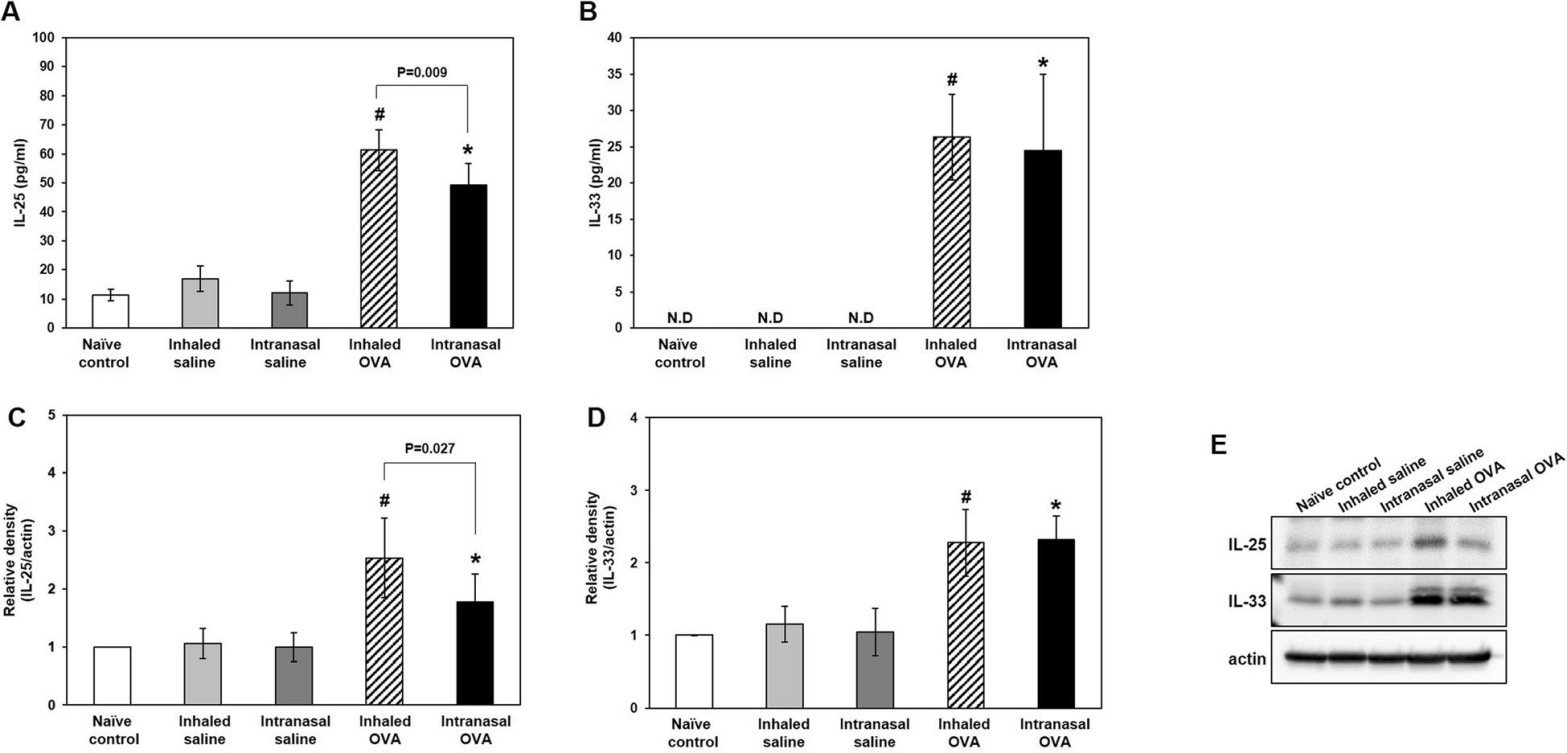
Epithelial cytokine levels in BALF and lung tissues. Enzyme immunoassay of IL-25 (**a**) and IL-33 (**b**) levels. Relative density (**c**, **d**) and representative western blots (**e**) of IL-25 and IL-33 proteins in control and OVA-sensitized and challenged mice. Bars represent the mean ± SD (*n* = 5/group). ^#^*p* < 0.05 vs. inhaled saline, ^*^p < 0.05 vs. intranasal saline

Epithelial cytokines released upon exposure to various allergens promote allergic asthma [17]. To assess whether the mode of OVA challenge influences epithelial damage, we evaluated the levels of the epithelial cytokines IL-25 and -33 in BALF (Fig. 8a, Fig. 8b) and lung tissues (Fig. 8c-e). Our results showed protein levels of IL-25 and -33 in BALF were upregulated in inhaled OVA group and intranasal OVA group as compared with inhaled saline group and intranasal saline group, respectively. IL-25 level in BALF was statistically higher by inhaled as compared to intranasal OVA delivery (*p* = 0.009, Fig. 8a). However, there was no difference in the level of IL-33 according to the OVA administration method in BALF (Fig. 8b). Consistent with results from BALF, western blot analysis (Fig. 8c, Fig. 8d) and representative images (Fig. 8e) showed that protein levels of IL-25 and -33 and in lung tissues were increased in inhaled OVA group and intranasal OVA group as compared with inhaled saline group and intranasal saline group, respectively. Lung IL-25 level was statistically higher by inhaled as compared to intranasal OVA delivery (*p* = 0.027, Fig. 8c). However, there was no difference in the level of IL-33 according to the OVA administration method (Fig. 8d).

## Discussion

In this study, we established mouse models of allergic asthma with different challenge routes and compared their phenotypes. Our results showed that typical asthmatic features including eosinophilic infiltration, AHR, mucus production, and elevated IgE level were represented in all OVA-sensitized and challenged mice. However, there were some notable differences between inhaled and intranasal OVA challenge, including the region and severity of lung inflammation and mucus secretion, as determined by histological analyses; AHR, which was enhanced to a greater extent in the inhaled as compared to the intranasal OVA group following OVA sensitization; and the levels of Th2 and epithelial cytokines, which were upregulated in the inhaled as compared to the intranasal OVA group. These findings indicate that the pathophysiologic pattern of asthma varies according to the mode of induction.

The major histopathological features of asthma include epithelial shedding, increased airway smooth muscle mass, mucus hypersecretion, subepithelial fibrosis, and inflammatory cell infiltration [22]. This airway inflammation and remodeling can occur in the central and small airways and lung parenchyma, resulting in lung hyperinflation due to extensive mucus plugging [23–26]. In this study, lung inflammation and mucus secretion were associated with an increase in relative lung weight in all OVA-sensitized and challenged mice. H&E staining revealed that OVA-sensitized mice challenged by inhalation exhibited pulmonary congestion and hemorrhage and eosinophilic infiltration in pulmonary vessels, alveolar ducts, and alveoli. In mice with intranasal OVA challenge, inflammation was mostly in peribronchial regions. Stronger PAS staining was observed in the bronchial epithelium of the intranasal as compared to the inhaled OVA group after OVA sensitization. Thus, the challenge route plays a critical role in the histological features of asthma, with inhalation and intranasal delivery of the allergen inducing asthma in the small and large airways, respectively. Our results suggest that animal models of asthma with different histopathological patterns can be established by varying the challenge route.

AHR, the most important symptom of asthma [22], is caused by increases in airway smooth muscle mass, mucus secretion, and inflammatory exudation [27]. Some in vivo studies have shown that prolonged allergen exposure causes structural changes in the airway and promotes inflammation through the interaction of various immune cells such as eosinophils, activated T lymphocytes, mast cells, and dendritic cells. In particular, Th2 cytokines including IL-4, -5, and -13 play critical roles in the development and maintenance of asthma. IL-4 regulates allergic inflammation by promoting Th2 cell differentiation, IgE synthesis, and mucus hypersecretion [28, 29]; IL-5 promotes eosinophilic inflammation and infiltration into the airways [30, 31]; and IL-13 induces AHR [32]. The results of our study showed that airway reactivity was enhanced by inhaled as compared to intranasal OVA challenge, with a corresponding upregulation in the levels of Th2 cytokines including IL-4, -5, and -13. Thus, Th2 cytokine-dependent airway reactivity after OVA sensitization is enhanced by OVA challenge via the inhaled as compared to the intranasal route, suggesting that the mode of challenge can differentially alter the physical features of the airways.

Airway epithelial cells are the first line of defense of the respiratory system against environmental stimuli [33]. However, in asthmatic patients, the barrier function of the epithelium is undermined by disruption of tight junctions in the central and small airways and lung parenchyma [34, 35]. The latter two produce more Th2 cytokines and chemokines involved in the initiation and development of inflammatory responses; moreover, inflammation at these distal sites is more severe than that in large airways. Damaged epithelial cells secrete the cytokines thymic stromal lymphopoietin and IL-25 and -33, which contribute to asthmatic features of the respiratory tract e.g., eosinophil infiltration, elevation in IgE level, increased mucus secretion, and epithelial cell hyperplasia/hypertrophy by stimulating the production of Th2 cytokines (IL-4, -5, and -13) in allergen-induced mice [36, 37]. Th2 cytokines are released in type 2 innate lymphoid cells (ILC2s) which are recruited by epithelial cell-derived IL-25 and -33 as well as in cluster of differentiation 4+ T lymphocytes [38, 39]. In addition, ILC2-derived Th2 cytokines exacerbate airway inflammation through subsequent adaptive T cell responses [18].

We measured the levels of epithelial cytokines and epithelial damage markers in lung tissue in our asthma models and found that consistent with our findings for Th2 cytokines, IL-25 and -33 levels in BALF and lung tissues were increased in two OVA challenge groups as compared to each control group. Especially, IL-25 protein in the lung were upregulated by inhaled as compared to intranasal OVA challenge with increased IL-13 protein in OVA-induced allergic asthma. Although IL-25 and IL-33 are implicated in promoting Th2-type immune responses, these cytokines belong to different cytokine families and have different roles in cellular sources and biological responses. IL-25 is a member of the IL-17 family and is produced by epithelial cells, Th2 cells, mast cells, macrophages, eosinophils and basophils. IL-25 signals through a heterodimeric complex of IL-17RA and IL-17RB, induces TRAF6-mediated activation of NF-κB, and produces mainly IL-5 and -13 from immune and structural cells. Also, IL-25 induces airway neutrophilia in a mouse model in an IL-17A-dependent manner [40–42]. In our results, IL-13 and IL-25 protein in the lung were upregulated by inhaled as compared to intranasal OVA challenge in OVA-induced allergic asthma. Moreover, OVA inhalation induced the significant increase of neutrophilic infiltration in lung. Thus, IL-25 epithelial cytokine-mediated asthmatic pathophysiology was more severe in AHR with companied with neutrophil infiltration by inhaled than by intranasal OVA delivery after OVA sensitization.

IL-33 is a member of the IL-1 cytokine family such as IL-1β and IL-18. IL-33 is abundantly expressed in endothelial cells, epithelial cells and fibroblast-like cells. While IL-33 belongs to the IL-1 family, the regulatory mechanisms for production and secretion of IL-33 are distinct form those for IL-1 β and IL-18. IL-33 is constitutively expressed and stored as a full IL-33 protein stores in the nucleus. Full length IL-33 has immunological activities and cleavage of IL-13 inactivates the cytokine [43]. Moreover, IL-33 receptor, ST2, is expressed on a wide variety of cell types including vascular endothelial cells, epithelial cells, eosinophils, and, other immune cells [44–46]. Therefore, IL-33 produced various cytokines beside IL-5 and IL-13 and has crucial roles in cellular injury, necrosis, and immune responses [47]. Currently, more studies are necessary to elucidate the functional roles of IL-33 and involved mechanisms during immune responses including asthma. Our results showed that IL-33 level in BALF and lung tissues were increased in two OVA challenge groups compare to each control group. However, there is no change according to OVA challenge mode. In our results, although IL-33 plays important role in induction of asthma, the mode of challenge was not affected in asthmatic patterns.

Although additional studies are needed to clarify the mechanistic basis for this observation, epithelial damage-mediated asthmatic pathophysiology was more severe by inhaled than by intranasal OVA delivery after OVA sensitization due to the larger diffusion area. Thus, epithelial cytokines released by damaged epithelial cell partly contribute to the pathophysiological patterns of asthma according to the mode of delivery after allergen sensitization.

## Conclusions

To understand complex asthma phenotypes, it is important to identify the best appropriate animal model. We compared asthmatic pathology induced by different challenge routes in murine models of allergic asthma. Our results showed that Th2 cytokines and AHR showed a greater increase by inhaled as compared to intranasal OVA challenge in OVA-sensitized mice. In particular, inhalation of OVA induced the expression of epithelial cell-derived cytokines to a greater extent than intranasal delivery. These different models can provide a better understanding of the pathophysiology of human asthma and can be useful for developing therapeutic targets for heterogeneous asthma phenotypes.

## Data Availability

The datasets used and/or analyzed during the current study are available from the corresponding author on reasonable request.

## Abbreviations

AHR: Airway hyperresponsiveness
BALF: Bronchoalveolar lavage fluid
H&E: Hematoxylin and eosin
IgE: Immunoglobulin E
IL: Interleukin
ILC2: Type 2 innate lymphoid cell
OVA: Ovalbumin
PAS: Periodic acid Schiff
Th2: T helper 2
